# Common molecular profile of multiple structurally distinct warfare arsenicals in causing cutaneous chemical vesicant injury

**DOI:** 10.1038/s41598-024-83513-1

**Published:** 2025-02-22

**Authors:** Ritesh Kumar Srivastava, Suhail Muzaffar, Jasim Khan, Mohit Bansal, Anupam Agarwal, Mohammad Athar

**Affiliations:** 1 UAB Research Center of Excellence in Arsenicals, Department of Dermatology, University of Alabama at Birmingham, Volker Hall – 509, 1670 University Blvd., Birmingham, AL 35294-0019 USA; 2 Department of Pathology, University of Alabama at Birmingham, Birmingham, AL USA; 3 Division of Nephrology, Department of Medicine, University of Alabama at Birmingham, Birmingham, AL USA

**Keywords:** Chemical warfare agents, Arsenicals, Skin injury, Stress signalling, Apoptosis

## Abstract

Skin exposure to arsenicals such as lewisite and phenylarsine oxide leads to severe cutaneous damage. Here, we characterized the molecular pathogenesis of skin injury caused by additionally structurally distinct warfare arsenicals including diphenylchlorarsine (DPCA), diphenylcyanoarsine (DPCYA), diethylchloroarsine (DECA). Cutaneous exposure to DPCA/DPCYA showed marked increase in skin erythema and edema at 6 and 24 h followed by scar formation at 72 h, while DECA did not produce such visual injuries in mouse skin. Clinical observations showed significant increase in Draize score and skin bi-fold thickness in a time-dependent manner. DPCA or DPCYA-exposed skin histology revealed highly inflamed hypodermal areas with infiltrated immune cells at 6 and 24 h, however, epidermal cell necrosis was seen at 72 h. Significantly high number of macrophage infiltration observed at 6 h, whereas peak neutrophil infiltration occurred at 72 h. Number of micro-blisters also increased. However, these effects were nonsignificant following topical DECA exposure. RT-PCR confirmed augmented inflammatory responses in the skin challenged with both DPCA/DPCYA, which accompanied increased ROS and unfolded protein response (UPR) signaling. DECA also increased ROS with changes in UPR. Disrupted tight (Yap/ZO-1) and adherens (Yap/α-Catenin) junction proteins underlie time-dependent apoptotic cell death of epidermal keratinocytes. Thus, these studies identify arsenicals-manifested signaling pathways similar to those of lewisite.

## Introduction

Arsenicals are highly reactive inorganic and organic derivatives of arsenic. Inorganic arsenicals are arsenic trioxide (As_2_O_3_) and arsine gas (AsH_3_) which have industrial importance. However, these inorganic arsenicals are toxic and may be fatal to humans if accidentally exposed in large concentrations^1–3^. On the other hand, due to extremely high toxicity and fast acting potential of organoarsenical compounds, they were specifically developed as chemical warfare agents (CWAs) during World War I and II (WWI/II) to be used against military personnel^4,5^. Syntheses of these chemicals are relatively easy as they can be produced using arsenic trichloride (AsCl_3_) as a precursor and replacing one or more chlorine atoms with an organic moiety, such as methyl, ethyl, phenyl, or their derivatives and can produce the desired arsenical^4,6^. After WWII, the unused arsenical CWAs were either stockpiled, buried, or dumped into different water bodies worldwide^7,8^. Some of these locations are known whereas others remain undescribed. Excavation of geographically unknown buried ammunition at the construction sites in many Asian and European countries led to accidental exposure of many laborers^9–11^. Likewise, corroded munitions loitering in the Baltic Sea are polluting sea water and exposing associated marine life^12,13^. Thus, both inorganic and organoarsenicals still pose a significant threat to environmental and human health.

**Fig. 1:**
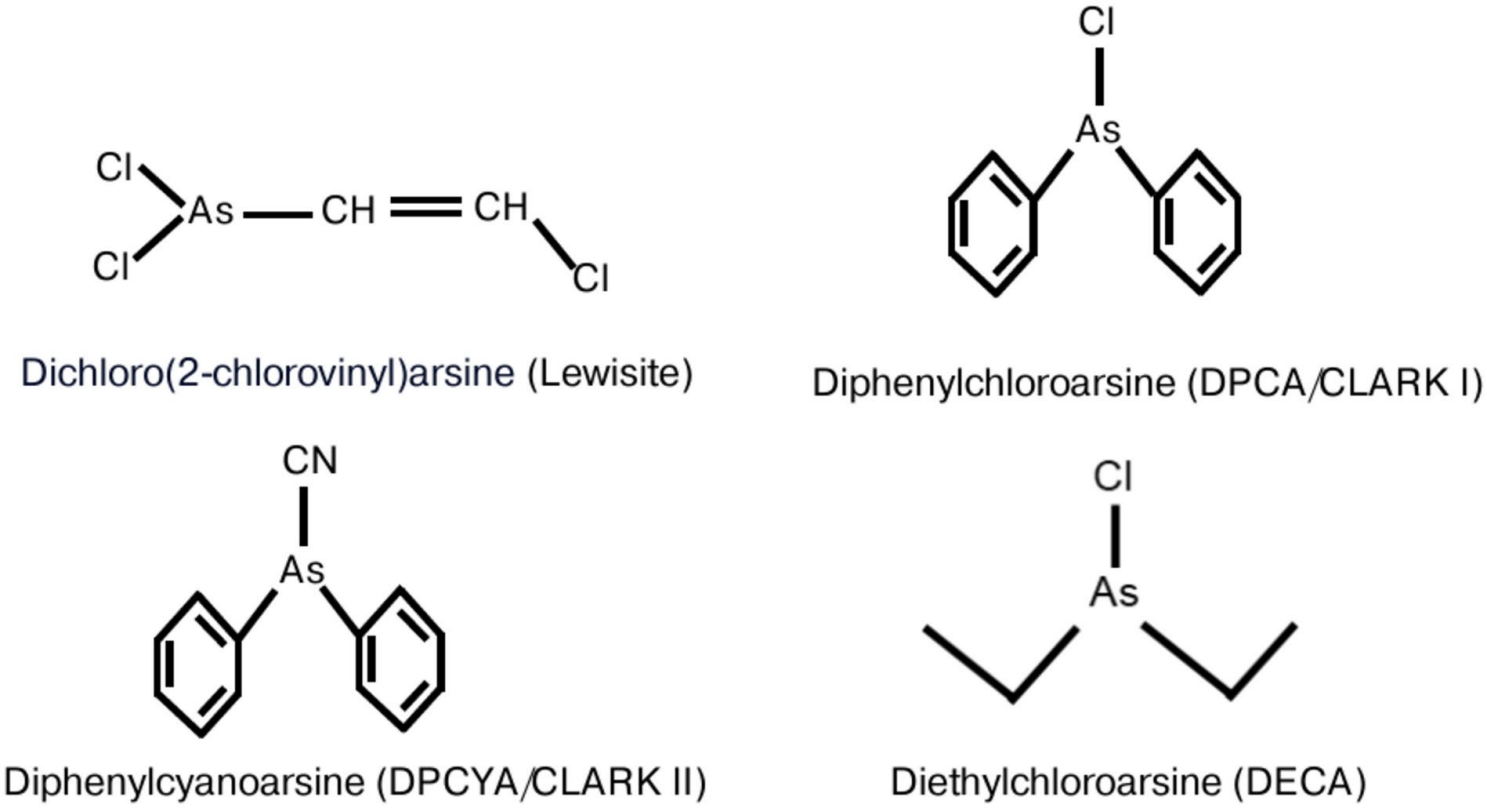
Chemical structure of organoarsenicals**.** Dichloro(2-chlorovinyl)arsine (lewisite), diphenylchloroarsine (DPCA), diphenylcyanoarsine (DPCYA), and diethylchloroarsine (DECA).

Several structurally related but distinct organoarsenicals such as lewisite, diphenylchloroarsine (DPCA), diphenylcyanoarsine (DPCYA), and diethylchloroarsine (DECA) (Fig. 1) are described as CWAs^14,15^. Out of these four arsenicals, lewisite became the lead candidate due to its rapid vesicating and highly toxic activities which can cause both cutaneous and systemic damage^16,17^. Earlier, we and others have shown that murine skin exposure to lewisite triggers skin tissue disruption characterized by increased erythema, edema, necrosis,  etc.^18–20^. We also observed that cutaneous lewisite exposure in mice leads to acute kidney injury (AKI) and acute lung injury (ALI)^21,22^. Similarly, neurological toxicity of lewisite mixture with other vesicants is also known^10^. However, very limited information is available regarding skin or systemic toxicity associated with DPCA, DPCYA, or DECA exposure. DPCA and DPCYA also known as Clark-I and Clark-II respectively, were given coded name as Doubled Blue Cross agents during WW-I by German forces^23,24^. These arsenicals were developed as sternutators to attack the nasal passage of the enemy personnel to induce vomiting, sneezing, and headache^25^. However, their toxicity profile may also include various additional characteristic such as skin and tissue irritant manifestations some of which are similar to lewisite. A few studies by our group and others demonstrated that exposure to DPCA is associated with AKI, pulmonary edema, and constrictive bronchiolitis in mice^21,26,27^. Due to its serious toxicity, DPCA was possibly used as a chemical weapon on the Western Front during the trench warfare of World War I^28^. In a study, the histological changes in the skin following DPCA and phenyldichloroarsine exposure in nude mice were characterized by the degeneration of epidermal cell nuclei, cytoplasmic vacuolization, and cleft formation within the basement membrane^29^.

DPCYA, a known successor of DPCA was discovered by the German scientists Sturniolo and Bellinzoni in 1981. The median incapacitating concentrations (ICt50) for DPCA and DPCYA have been estimated as 12 mg-min/m and 30 mg-min/m respectively^30^. DCPYA and its derivative are known to induce cellular toxicity^14,31^. Interestingly, the degradation products of these two arsenicals are also found to be very toxic in nature. Diphenylarsinic acid (DPAA), an intermediate metabolite of DPCA, and DPCYA, has been reported to cause cerebellar symptoms among the residents of Kamisu, Japan. These included mental retardation and associated brain atrophy ^24^. In an in vitro study, DPAA showed both cytotoxicity and genotoxicity in HepG2 and V79 Chinese hamster cells^32^. A study in a murine model showed that oral administration of DPAA induces oxidative and nitrosative stress in cerebellar Purkinje cells, which may lead to cerebellar symptoms in these exposed animals^33^. We have summarized the effects of DPAA in human and experimental models in a recent publication^34^.

DECA is a less stable organoarsenical that can be generated by heating Ethyldichloroarsine (ED) at 120^0^C in the presence of tetraethyllead^35^. ED is highly toxic and may be fatal if inhaled, ingested, or absorbed through the skin^16^. Liquid ED can induce lacrimation, pulmonary edema, and blistering in the skin^36^.

Our previous studies in both in vitro and in vivo model systems revealed the pathogenesis of cutaneous inflammation and tissue damage by lewisite and PAO. We showed that their molecular pathogenesis involved the induction of oxidative stress and endoplasmic reticulum (ER) stress, which lead to the accumulation of the unfolded proteins and activation of the unfolded protein response (UPR) signaling pathway^20,37^. During ER stress, an ER-resident protein kinase, PERK phosphorylates the eukaryotic translation initiation factor eIF2α, thereby enhancing the expression of the transcription factor ATF4^38^. ATF4 triggers the expression of CHOP, a transcription factor that promotes the expression of genes associated with stress-induced cell death^39^. Our earlier studies highlighted a central role of CHOP in PAO-induced apoptosis and cytokine production in human skin keratinocytes^37^. We also demonstrated that inorganic arsenicals such as arsenic trioxide (ATO)-induced UPR signaling pathway leads to impairment in macrophage functions and blockade of this signaling by a chemical chaperon, 4-phenylbutyric acid (4-PBA) attenuated ATO-induced expression of UPR proteins as well as restoration of macrophage functions^40^. Protective effects of 4-PBA have also been demonstrated against PAO and lewisite induced skin inflammation, micro-blistering and tissue damage in murine models^20,37^.

Although distinct pathological manifestations and clinical outcomes have been demonstrated by various structurally distinct warfare arsenicals, the molecular mechanism involved in their pathophysiology remains unclear. A prominent utility of this understanding of the molecular mechanisms of these chemicals is to develop an effective medical countermeasure (MCM) to attenuate their toxicity. In this regard, an important need is to identify druggable molecular targets that are common among various toxic chemicals. Thus, an MCM so developed may be effective against multiple toxic chemicals. Therefore, this study is designed to investigate whether structurally related distinct arsenicals manifest some similar toxic effects to those induced by lewisite. Potential mechanisms of skin inflammatory and cell death responses were also investigated.

## Materials and methods

### Animal studies

All of the animal experimental protocols were approved by the Institutional Animal Care and Use Committee (IACUC) of the University of Alabama at Birmingham and MRIGlobal. We confirmed that all methods were carried out in accordance with relevant guidelines and regulations. The present study is reported in accordance with ARRIVE guidelines (https://arriveguidelines.org) for the reporting of animal experiments. Animals used in this study were housed in pathogen free, temperature (70–72^0^ F) and humidity (40–60%) controlled facility with free access to food and water. Before starting the experiment, mice were randomized and assigned to each group of arsenical exposure. Buprenorphine (0.05–0.10 mg/kg) was administered 30 min before anesthesia for pain management. At the time of cutaneous arsenical exposure, mice were anesthetized with ketamine (100 mg/kg) and xylazine (5 mg/kg) by intraperitoneal injections. Ptch1^+/-^/SKH-1 hairless mice (N = 6/group) 10–12 weeks old male and female (equal number) were topically challenged to ethanol diluted DPCA, DPCYA and DECA at doses 6.381, 6.154 and 4.064 mg/kg respectively on dorsal side of the skin (2X4 cm^2^) and observed for 6 h, 24 h and 72 h. A group of control mice were identically challenged with vehicle (ethanol) alone. Earlier, we have screened multiple murine models (C57/BL/6, FVB, SKH-1 and Ptch1^+/-^/SKH1) of chemical cutaneous injury and demonstrated that Ptch1^+/-^/SKH-1 mouse is highly sensitive to these chemicals^20,41^. Thus, we employed this mouse strain to further study the effects of multiple other structurally different arsenicals. In this study, the dose selection of DPCA, DPCYA, and DECA was based on our previous published studies, where we selected a dose of lewisite (5 mg/kg) from a dose escalation study. This dose of lewisite produced cutaneous inflammatory and tissue damage responses in murine skin that are considered as median^19,41^. Therefore, to compare the cutaneous toxicity of these arsenicals, we used their molar equivalent dose to 5 mg/kg dose of lewisite. Similarly, time-point selection in this study is based on our observations where lewisite produces early inflammatory cytokine production at 6 h and late tissue damage responses at 24 and 72 hr^19^. Animal exposure studies to arsenicals, DPCA, DPCYA, and DECA were performed at MRIGlobal (Kansas City, MO, USA)^20^. MRIGlobal is a certified facility and has vast experience in conducting experimental studies using highly toxic chemicals including CWAs. These chemical agents are restricted to use, synthesize and store in general laboratory settings. Therefore, all the war grade chemicals used in this study were synthesized in limited quantity and stored by MRIGlobal, Kansas City, employing all the required safety procedure. Purity of each chemical was tested by MRIGlobal using Gas chromatography with flame ionization detection (GC-FID) technique. The results showed that DPCA, DPCYA, and DECA were 98.7%, 99.8% and 95.6% pure respectively. (Certificate of analysis attached as supplementary Fig. S1, S2 and S3). Following chemical exposure, skin injuries were photographed and evaluated via modified Draize scores (erythema score + edema score + necrosis score) as described previously^18^. Briefly, mouse skin showing minor edema, minor erythema, and minor/focal necrosis were scored 1 for each parameter, whereas mice with moderate edema and erythema together with early sign of necrosis (25%–50% of skin area is necrotic) were scored 2 for each parameter. Severe edema, erythema, but moderate necrosis (50%–75% of skin area is necrotic) scored 3; and mice skin with severe necrosis (75%–100% of skin area is necrotic) scored as 4. Bifold thickness of the skin was measured with an electronic digital caliper and presented in millimeter (mm). The skin samples were collected and snap frozen in liquid nitrogen or fixed in 10% formalin for further analysis.

### Histological evaluations

Hematoxylin and eosin (H&E) staining of control and arsenical-treated mouse skin sections was performed as described earlier^41^. Paraffin-embedded skin sections on glass slides were deparaffinized in xylene and hydrated through graded ethanol and water. The rehydrated skin sections were stained with H&E stains. After staining, the skin sections were examined under a brightfield microscope for observation of infiltration of inflammatory cells and epidermal and dermal separation (micro-vesication). At least 3–5 skin sections were analyzed from each group and multiple images were captured from each skin section for quantitative analysis.

### Immunohistochemistry

Immunohistochemistry (IHC) of skin sections from control and arsenical-treated mouse skin was performed according to the protocol described previously^42^. Briefly, the glass slide–adhered skin Sects. (5 μm) from various treatment groups were deparaffinized in xylene, rehydrated in descending alcohol concentrations and water, and then incubated with antigen-unmasking solution according to the manufacturer’s protocol (Vector Laboratories, CA, USA). This was followed by incubating the skin sections in blocking buffer (1% BSA with 4% goat serum in PBS) for 30 min at 37 °C. After the blocking step, the primary antibodies against F4/80 (Abcam, Cambridge, MA, USA; ab100790) and MPO (Abcam; ab208670) were added, and the slides were incubated at 4 °C overnight. The following day, a universal peroxidase-coupled secondary antibody was added, and the tissue was washed with PBS before exposure to a substrate of 0.1% 3-3ʺ-diaminobenzidine-tetrahydrochloride (DAB; Sigma, St. Louis, Mo., USA). These stained sections were examined under the Keyence fluorescence microscope (model BZ-X710; Osaka, Japan). Multiple images from 4–5 individual skin sections per group were captured for quantitative analysis.

### Immunofluorescence staining

For immunofluorescence staining, the deparaffinized skin sections attached to glass slides were incubated with antigen unmasking solution (Vector Laboratories, Burlingame, CA, USA) according to the manufacturer’s instructions. Following unmasking, the skin sections were incubated with the blocking buffer as described for IHC in 1 × PBS for 30 min at 37 °C. Primary antibodies, YAP (Santa Cruz Biotechnology, Dallas, TX, USA; sc-101199), α E-catenin (Santa Cruz Biotechnology; sc-9988) and ZO-1 (Santa Cruz Biotechnology; sc-33725) were added to the skin sections and incubated at 4 °C overnight. The next day, fluorescence-coupled secondary antibodies were added, the sections were washed with PBS and mounted with DAPI. These mounted skin sections were analyzed to observe the co-localization and disruption of tight (Yap/ZO-1) and adherens (Yap/α-Catenin) junction proteins under the fluorescence microscope.

### Reactive oxygen species (ROS) quantification

Quantitative ROS analysis of control and arsenical-treated mouse skin lysates was carried out using the OxiSelect™ in vitro ROS Assay Kit (Cell Biolabs, San Diego, CA, USA) according to the manufacturer’s instructions. Briefly, mouse skin (50–60 mg) was homogenized in 0.5 ml PBS using the Power Gen 1000 homogenizer. After homogenization, skin lysates were centrifuged at 14,000 × *g* for 10 min, and 25 μl fat-free clear supernatant was used for ROS quantification as also demonstrated earlier^41^. The fluorescence intensity was recorded at 485/528 nm excitation/emission wavelength using the plate reader (Synergy Neo2 Multimode Reader, BioTek). Data were presented as % change of the mean of fluorescence intensity ± SEM.

### nanoString analysis

nanoString analysis for inflammation-related genes was performed as described previously^41,43^. The process involves the hybridization of target mRNA with gene-specific probe pairs followed by fully automated post-hybridization processing and data acquisition. Briefly, RNA from the skin of arsenical treated and control mice was hybridized with the nanoString nCounter mouse Inflammation V2 panel (nanoString Technologies) at 65 °C overnight. Hybridized samples immobilized onto the nCounter cartridge were imaged on nCounter SPRINT Profiler (nanoString Technologies) as described earlier. We analyzed data with the help of a nCounter nSolver platform (nanoString Technologies). A fold-change cutoff of ± 1.5 and Padj < 0.05 was set to commonly upregulated or downregulated inflammatory genes.

### RT-PCR analysis

Real time PCR (RT-PCR) was done on a 7500 fast real-time PCR system (Applied Biosystems, Foster City, CA, USA) using TaqMan qPCR Master Mix and TaqMan primers. One microgram (1 µg) RNA was converted into first-strand cDNA using iScript cDNA synthesis kit (Bio-Rad Laboratories, Hercules, CA, USA) For each group, at least 3 individual skin samples were used for quantitative mRNA analysis after normalization to an endogenous control β-actin. Relative fold-changes in gene expression were calculated and presented as fold change in gene expression.

### Western blot analysis

Protein lysates were prepared from mouse skin tissues in ice-cold RIPA lysis buffer (Santa Cruz Biotechnology) using the Power Gen 1000 homogenizer (Fisher Scientific, Pittsburgh, PA, USA). Protein concentration was measured using a Bio-Rad protein assay DC kit (Bio-Rad Laboratories) according to the manufacturer’s instructions. 30 µg protein from each sample was resolved by Sodium dodecyl-sulfate polyacrylamide gel electrophoresis (SDS-PAGE), transferred to the polyvinylidene difluoride (PVDF) membrane which were then blocked with 5% nonfat dry milk for 1 h and probed with primary antibodies (1:1000 dilution) overnight at 4 °C. Some of the membranes were cut prior to probing with antibodies to see the expression of loading control β-actin and the required protein separately on the same blot. The following antibodies were used in this study: ATF4 (Invitrogen, MA5-32,364), CHOP (Cell Signaling, 2895), cleaved Caspase 3 (Novus Biologicals, NB100-56,708) and HRP conjugated β-actin antibody (Cell Signaling, 12,620). The following day, the membrane was washed with Tris-buffered saline/Tween 20 (TBST) buffer three times. After washing with TBST, the membranes were incubated with horseradish peroxidase (HRP) conjugated secondary antibody for 2 h at room temperature. After washing with TBST buffer three times, protein bands were visualized using autoradiography film (Densville Scientific Inc.) or with an iBright1000 imaging system (Thermo Fisher Scientific, MA, USA) using enhanced chemiluminescence detection reagent according to the manufacturer’s instructions (Santa Cruz Biotechnology). ImageJ scientific software was used for densitometric analysis of the bands.

### Terminal deoxynucleotidyl transferase dUTP nick end labeling (TUNEL) Assay

TUNEL assay was performed using the In-Situ Cell Death Detection Kit, Fluorescein (Roche Diagnostics, Indianapolis, IN, USA) according to the manufacturer’s instructions. Briefly, skin sections obtained from control and arsenical-treated mice were incubated with proteinase K (10–20 mg/ml in 10 mM Tris/HCL, pH 7.4) for 15 min. The sections were washed with PBS three times and incubated with the TUNEL reaction mixture for 1 h at 37^0^C. After washing with PBS, the skin sections were mounted with antifade DAPI before covering with a glass coverslip. The stained sections were visualized under a Keyence Fluorescence Microscope for TUNEL–positive (green fluorescence) cells. Multiple images were captured from different sections of each group. Keyence Microscope inbuilt software (Model BZ-X710, KEYENCE, Osaka, Japan) was used for quantitative analysis of TUNEL-positive cells.

### Statistical analysis

GraphPad Prism (GraphPad Software, Inc.) was used for statistical analysis. Data was represented as the mean ± standard error of the mean (SEM) of at least 3–6 individual mice from each group. Normalized data were analyzed by one-way or two-way analysis of variance (ANOVA). At least p < 0.05 was reported as significant.

## Results

### Characterization of skin injury caused by structurally different arsenicals

**Fig. 2:**
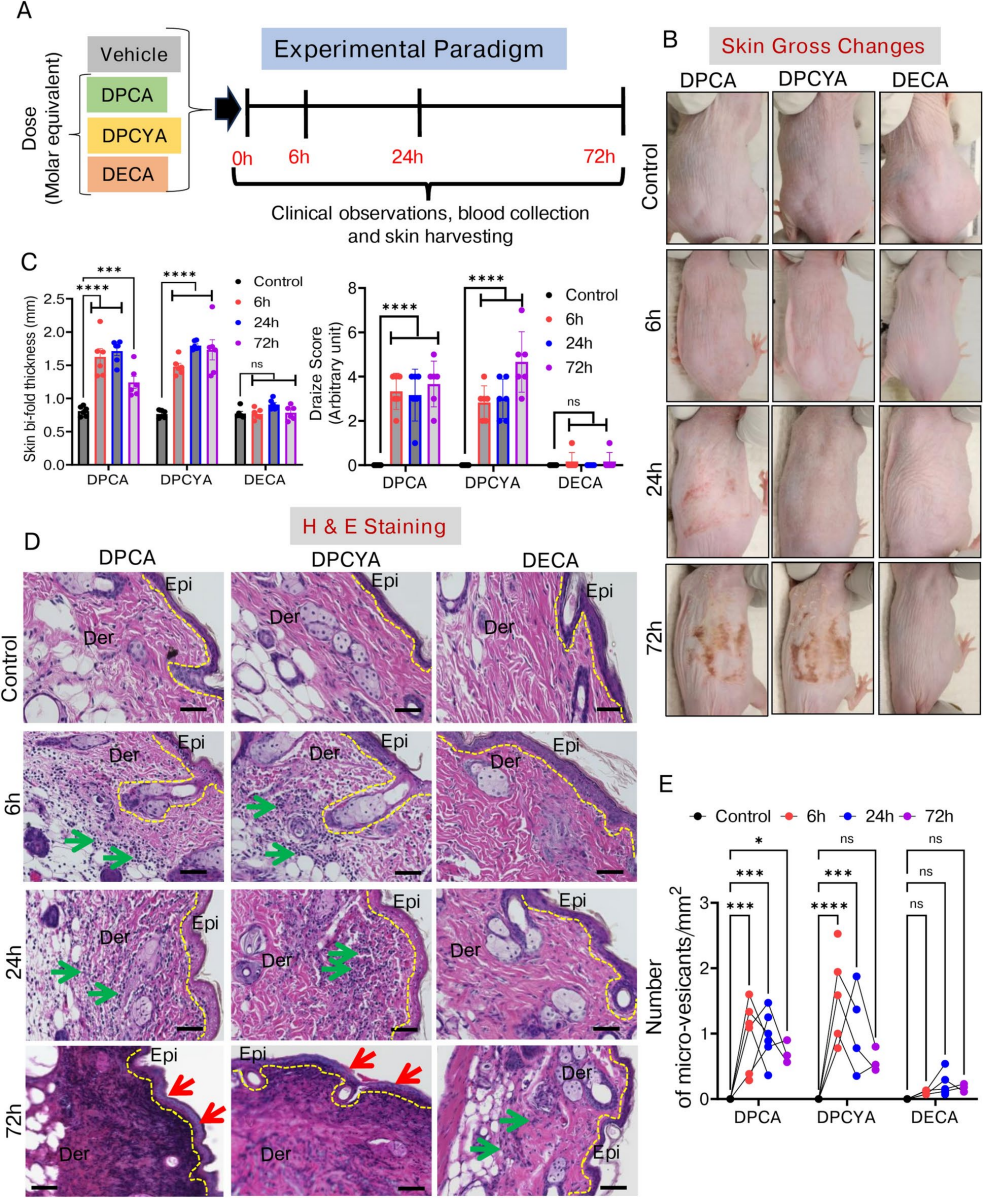
Time-dependent effects of structurally different arsenicals on cutaneous injury in Ptch1^+/-^/SKH1 hairless mice model. (**A**) The experimental design depicts the timeline of cutaneous arsenical exposures, clinical observations and skin sample collection. (**B**) Representative images showing gross skin injury effects with time following DPCA, DPCYA, or DECA exposure in mice. (**C**) Clinical scores, including measurements of skin bifold thickness and Draize score, were documented after 6 h, 24 h, and 72 h of arsenical exposure. (**D**) H&E staining of skin sections from the control and arsenicals exposed mice showing the infiltration of inflammatory cells (green arrows) and complete loss of epidermal cells (red arrows). Epi- Epidermis, Der-Dermis, Scale bar- 50 µm. (**E**) Histograms showing the average number of micro-vesicants/unit area of the skin sections. epidermal-dermal separation was recorded to count micro-vesication/slide sections at 200 × magnification. *P < 0.05, ***P < 0.001, ****P < 0.0001 showing significance compared to vehicle treated controls. ns-non-significant. N = 4–6/group.

As described in experimental design earlier, the dose of DPCA, DPCYA and DECA used in this study was based on the calculation of molar equivalent dose 5 mg/kg of lewisite. As depicted in Fig. 2A**,** the dorsal skin of Ptch1^+/-^/SKH-1 mice was topically administered with 6.381, 6.154 and 4.064 mg/kg dose of DPCA, DPCYA, and DECA respectively to study their time-dependent effects. We observed that following a single cutaneous application of DPCA and DPCYA, the exposed skin of mice became thick, and wrinkles appeared at 6 h and 24 h. After 72 h, cutaneous scar was visible in DPCA and DPCYA-challenged mice, however, no such effect was observed in DECA-challenged experimental animals even up to 72 h **(**Fig. 2B**)**. In DPCA and DPCYA-challenged mice, the skin bifold thickness and Draize score were significantly increased with time **(**Fig. 2C**)**. These progressive changes were observed beginning 6 h after DPCA and DPCYA exposure and remained significantly elevated till 72 h of the observation period. However, the changes in skin bifold thickness and Draize score in DECA-challenged mice were non-significant. H&E-stained skin sections from DPCA and DPCYA-challenged mice showed vast infiltration of inflammatory cells at 6 h and 24 h **(**Fig. 2D**, indicated by green arrows)**. At 72 h, a complete loss of epidermal cells in necrotic area of skin tissue was spotted throughout the skin sections **(**Fig. 2D**, indicated by red arrows).** In contrast, consistent with earlier observations, DECA-challenged mice showed non-significant changes in tissue morphology and immune cells infiltration compared to vehicle-treated control animals. Furthermore, the incidence of appearance of micro-vesicants (MV) (characterized by epidermal and dermal separation) was significantly higher at 6 h and 24 h after exposure compared to changes in 72 h exposure group **(**Fig. 2E**).** The prevalence of MV formation in DECA-challenged mice was not evident. Since arsenicals contain arsenic and skin absorption may cause systemic circulation and metabolism of these agents. Therefore, blood arsenic may predict their systemic exposure and toxicity. Previously, using Inductively Coupled Plasma Mass Spectrometry (ICP-MS), we measured blood arsenic levels in arsenicals-challenged mice^21^. These data demonstrated significantly increased blood arsenic levels only in DPCA and DPCYA-challenged mice, but not in DECA exposed mice, suggesting the poor cutaneous absorption and bioavailability for systemic circulation of DECA. These data also provide rationale for substantial higher cutaneous and systemic toxicity of DPCA and DPCYA as compared to DECA.

### Characterization of immune cells infiltration in the skin of arsenicals challenged mice

**Fig. 3:**
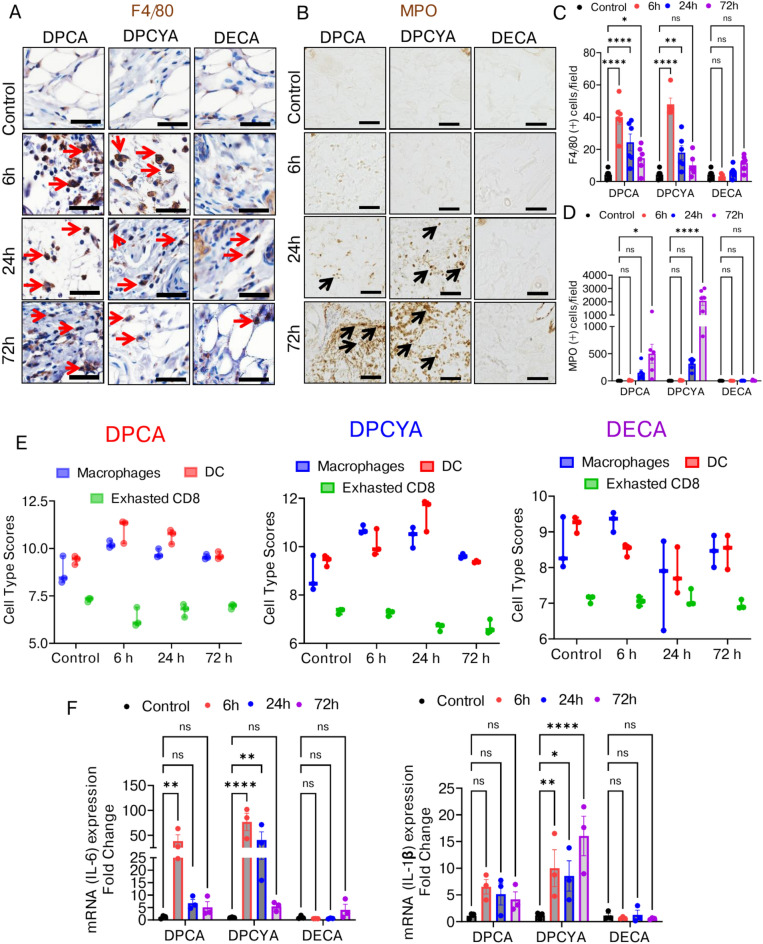
Effects of structurally different arsenicals on inflammatory response in the mouse skin.(**A**) Immunohistochemistry of skin sections showed upregulation of macrophage marker F4/80 (brown color-stained cells, red arrows) in the dermis after DPCA and DPCYA exposure. Scale bar-50 µm. (**B**) Neutrophil biomarker (MPO) (brown color-stained cells, black arrows) also showed elevated expression in skin treated with DPCA and DPCYA. Scale bar-50 µm. (**C**) Quantitative analysis of F4/80 positive cells indicated significantly higher macrophage infiltration in DPCA and DPCYA group. However, no significant changes observed in DECA group. (**D**) The histogram represents a time-dependent progressive increase in neutrophil infiltration in the skin of DPCA and DPCYA-challenged mice. No Significant changes were observed in DECA group. (**E**) Immune cell pathway score from nanoString analysis shows the relative abundance of macrophages, dendritic cells (DC), and exhausted CD8 + T cells with time after DPCA, DPCYA, and DECA exposure in mice. (**F**) RT-PCR analysis showing the relative mRNA levels of IL6 and IL-1β inflammatory genes in the skin of mice exposed to DPCA, DPCYA, and DECA. *P < 0.05, **P < 0.01, ****P < 0.0001 showing significance compared to vehicle treated controls. ns-non-significant. N = 4–6/group.

We characterized the immune cell infiltration in the DPCA, DPCYA, and DECA-challenged murine skin. IHC staining for the F4/80 (macrophage biomarker) showed that a significantly large population among the infiltrated immune cells were macrophages in the dermal region **(**Fig. 3A**).** Quantitative analysis revealed that the infiltration of macrophages in DPCA and DPCYA-treated skin peaked at 6 h, with a subsequent progressive decrease observed at 24 h and 72 h **(**Fig. 3C**).** The myeloperoxidase (MPO, a neutrophil biomarker) skin staining showed that DPCA and DPCYA exposure also leads to neutrophil infiltration **(**Fig. 3B**).** Surprisingly, the neutrophil infiltration was notably higher in the 72-h exposure group compared to the early time points (6 h and 24 h) **(**Fig. 3D**).** The DECA-challenged murine skin however did not show any significant infiltration of either macrophages or neutrophils **(**Fig. 3C and 3D**).**

Next, we used nanoString data to quantify the relative abundance of different immune cells in control and arsenical treated skin samples. Based on the raw scores of various immune cells, we identified that the overall abundance of macrophages and dendritic cells was higher in the DPCA and DPCYA-treated mice group compared to the control group **(**Fig. 3E**)**. On the other hand, the abundance of exhausted CD8 + T cells was reduced. While no significant changes were observed in abundance of any of these infiltrated cell types in DECA-challenged mice **(**Fig. 3E**).** Subsequently, we aimed to investigate whether exposure to these arsenicals augmented the expression of proinflammatory cytokines in the skin. RT-PCR analysis showed a significant increase in Il6 mRNA levels after 6 h and 24 h of skin exposure to DPCA and DPCYA **(**Fig. 3F**),** however, the expression levels decreased at 72-h post-challenge. Similarly, mice exposed to DPCYA showed markedly elevated *IL1β* levels after 6 h, 24 h, and 72 h **(**Fig. 3F**).** On the contrary, no significant changes in the levels of proinflammatory cytokines *IL6* and *IL1β* were observed in the mRNA expression of DECA-challenged mice.

### Expression profile of inflammatory genes by structurally different arsenicals

**Fig. 4:**
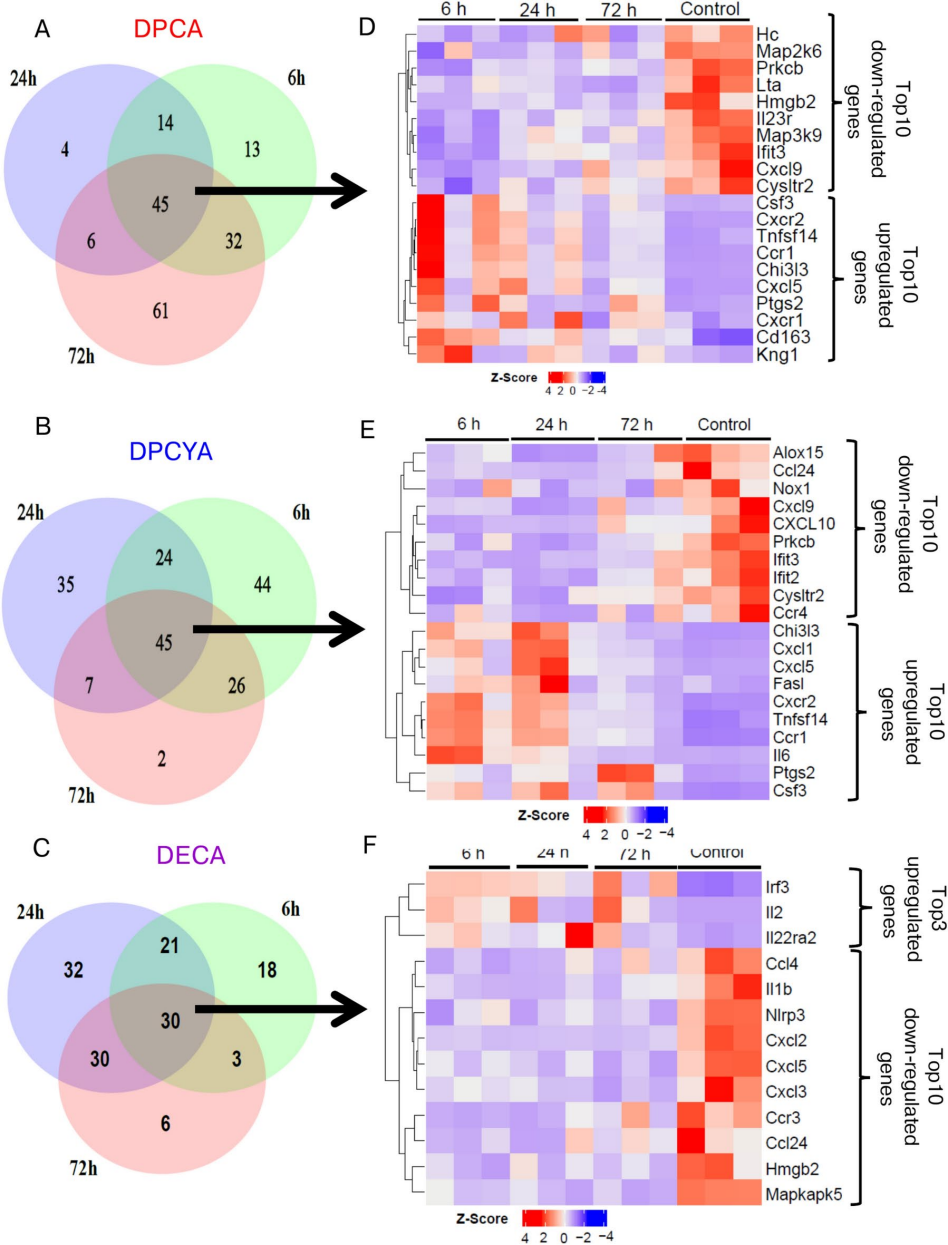

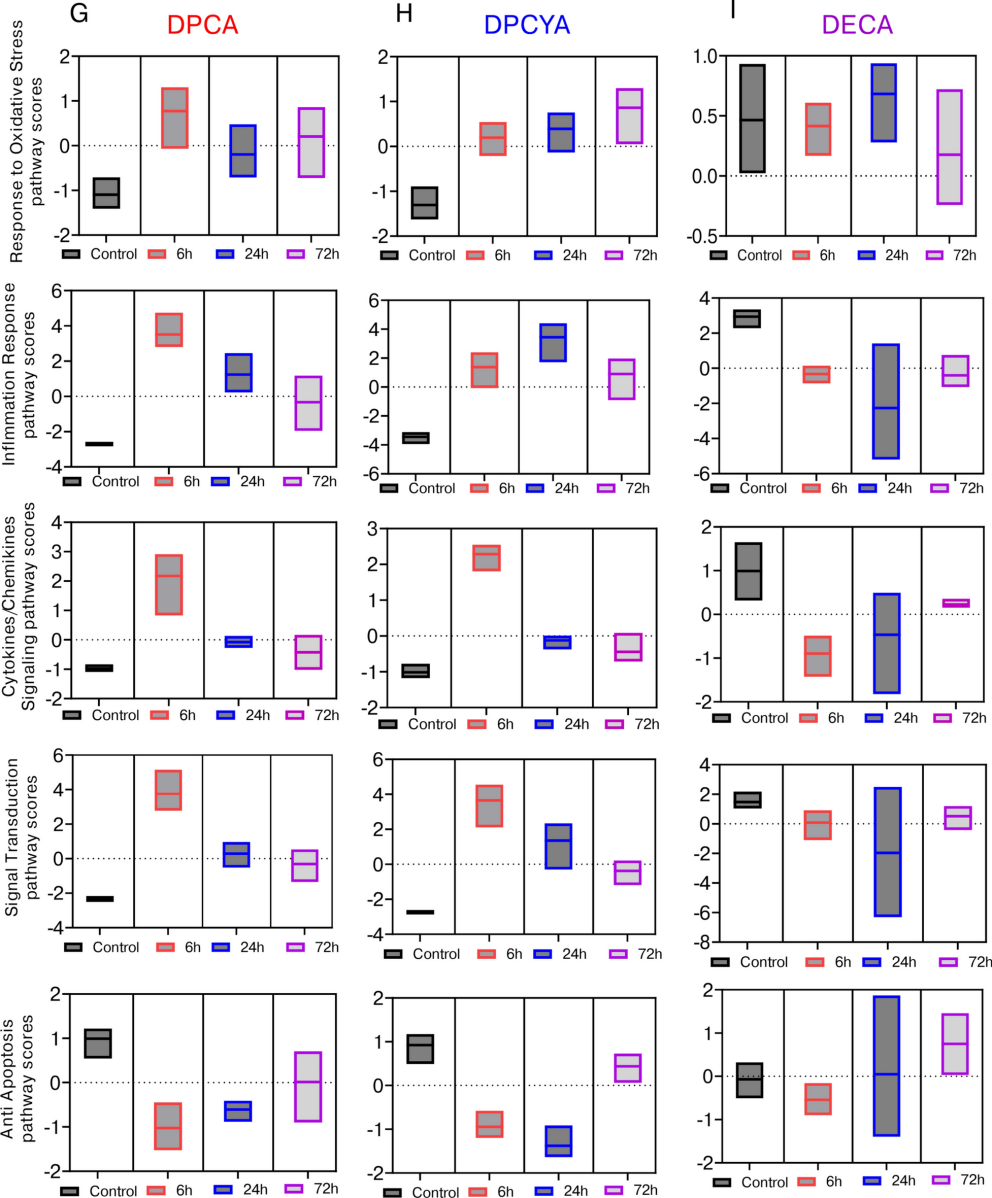
Expression profile of inflammatory genes in the murine skin following DPCA, DPCYA and DECA exposure. (**A, B and C**) Venn diagram showing number of commonly and uniquely altered inflammatory genes (FC ≥ 1.5 and P < 0.05) by DPCA, DPCYA or DECA exposure in murine skin at 6 h, 24 h and 72 h time-points. (**D, E and F**) Heat map showing cluster analysis of top 10 up and top 10 down-regulated inflammatory genes obtained from commonly altered genes at all the time points. Commonly altered genes (FC ≥ 1.5 and P < 0.05) at 6 h, 24 h and 72 h exposure contains 45 genes for DPCA, 45 genes for DPCYA and 30 genes for DECA. (**G, H and I**) Pathway scores, obtained from nanoString analysis, showing effects of DPCA, DPCYA and DECA on various cellular responses with respect to time.

To characterize the DPCA, DPCYA and DECA-induced cutaneous inflammation, we performed nanoString analysis of inflammatory genes in the skin samples. nanoString assay contained a panel of 254 inflammatory genes from mouse Inflammation V2 panel (nanoString Technologies). Venn diagram showing analysis of these genes identified that out of 254 genes, 45 inflammatory genes were commonly altered by DPCA and DPCYA, while 30 genes were commonly altered by DECA-challenge in the mouse skin compared to vehicle control (FC ≥ 1.5 and P < 0.05) among 6 h, 24 h and 72 h time-points. **(**Fig. 4A, 4B and 4C**).** Out of 45 commonly altered genes by DPCA and DPCYA, we identified top10 up and top10 downregulated genes as shown in Fig, 4D and 4E. These data showed that *Csf3, Cxcr2, Tnfsf14, Ccr1, Chi3l3, Cxcl5*, and *Ptgs2* were commonly upregulated and *Cxcl9, Cysltr2, Ifit3* and *Prkcb* were commonly downregulated between these two arsenicals. Conversely, *Kng1, Cd163* and *Cxcr1* were uniquely upregulated by DPCA, whereas *Cxcl1, Il6, Fasl* and *Cxcr2* were uniquely upregulated by DPCYA (Fig. 4D and 4E**)**. *Kng1* encode high molecular weight kininogen proteins, and evidence suggest that KNG1 play a significant role in inflammation, coagulation and apoptosis^44–46^. While Cxcr1 act as a receptor for interleukin 8, a chemotactic factor for neutrophils and can induce immediate cytotoxicity^47,48^. On the other hand, out of 30 commonly altered genes by DECA, only 3 were upregulated (*Irf3, Il2* and *Il22ra2*), while other top10 genes (FC ≥ 1.5 and P < 0.05) were downregulated **(**Fig. 4F**).** Next, we identified many common cellular responses for which both DPCA and DPCYA showed an increased pathway score (with respect to their skin exposure time) compared to vehicle-treated skin from control animals. These pathways include response to oxidative stress, inflammation, cytokine/chemokines signaling and signal transduction **(**Fig. 4G and 4H**)**. In contrast, anti-apoptosis pathway score was decreased among these two arsenicals **(**Fig. 4G and 4H**)**. DECA again did not show significant changes in the scores of these cellular responses **(**Fig. 4I**)**.

### Arsenicals-mediated disruption of tight and adherens junction is associated with enhanced oxidative and endoplasmic reticulum (ER) stress

**Fig. 5:**
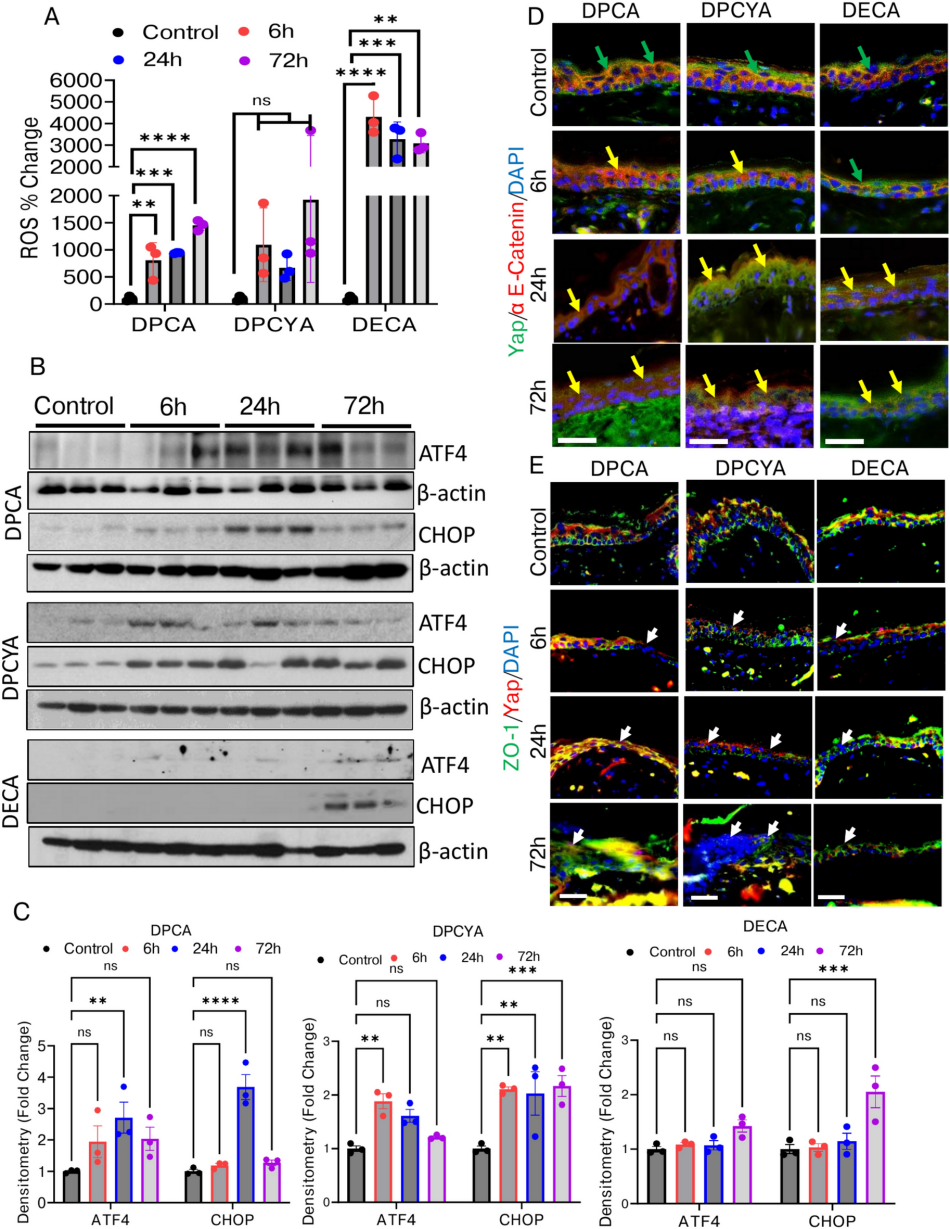
Effects of structurally different arsenicals on reactive oxygen species generation, UPR signaling proteins and disruptions of tight and adherens junction. (**A**) OxiSelect ROS detection assay showed increased ROS levels in the skin of DPCA, DPCYA, and DECA-challenged mice after 6 h, 24 h, and 72 h. (**B**) Western blot analysis of skin lysates collected at 6, 24 h and 72 h time-points showed elevated expression of ATF4 and CHOP following arsenical exposure. β-actin was used as an endogenous control. (**C**) Histogram representing densitometry analysis of western blots band intensity. (Also see supplementary fig. S4, S5 and S6 for full images of immunoblots). (**D**) Immunofluorescence staining of Yap/α E-catenin showing disruption of adherens junctions in the arsenical exposed skin. Green arrows representing co-localization of Yap/α E-catenin, while yellow arrows representing disruption and loss of these proteins in mouse epidermis with significant changes at 24 and 72 h. Scale bar-50 µm. (**E**) ZO1/Yap IF shows diffuse staining of tight junction proteins following arsenical exposure. Disruptions of tight and adherens junction proteins in the epidermal layer of skin could be evident at 24 and 72 h (white arrows). Scale bar-50 µm. *P < 0.05, **P < 0.01, ***P < 0.001, ****P < 0.0001 showing significance compared to vehicle treated controls. ns-non-significant. N = 3/group.

Earlier, we have reported that lewisite and PAO challenge to mouse skin enhanced ROS production^37,41^. Therefore, we also investigated whether exposure to DPCA, DPCYA, and DECA leads to enhanced ROS production compared to vehicle-treated controls. As detected by the OxiSelect ROS detection kit, significantly enhanced ROS levels were recorded in the skin of DPCA or DPCYA-treated mice after 6 h, 24 h, and 72 h **(**Fig. 5A**).** Surprisingly, DECA also induced significantly high ROS and that was maximum among the three arsenicals **(**Fig. 5A**).** Oxidative stress can disturb protein folding at the ER and thereby leading to protein misfolding and exacerbated UPR signaling pathway^49^. We investigated whether cutaneous exposure to DPCA, DPCYA, or DECA leads to ER stress in mice. Western blot analysis confirmed the upregulation of UPR signaling transcription factors ATF4 and CHOP in the skin of DPCA, DPCYA and DECA-challenged mice. Significant upregulation of ATF4 and CHOP was observed at 6 h, 24 h, and 72 h following exposure with DPCA and DPCYA, while DECA was able to induce these proteins only at 72 h **(**Fig. 5B, 5C** and Supplementary Fig. S4, S5 and S6)**. Intercellular junctions like adherens and tight junctions play a vital role in preserving the adhesion and barrier function of the skin epithelial cells^50^. Therefore, we examined the effects of DPCA, DPCYA, and DECA exposure on the adherens junction-associated proteins (Yap and α E-catenin) and tight junction-associated proteins (ZO-1) in mice. Immunofluorescence staining demonstrated Yap and α-catenin nicely co-localized in vehicle-treated control mice. However, DPCA and DPCYA exposure disrupted the expression and membrane localization of these proteins in the exposed skin starting at 6 h with significant changes at 24 and 72 h **(**Fig. 5D**).** Similarly, exposure to DPCA and DPCYA disrupted the expression and localization of ZO-1/YAP in mouse skin **(**Fig. 5E**).** The effect was minimal at all the time-points for both tight and adherence proteins in DECA-challenged mice **(**Fig. 5D and 5E**).**

### Arsenical exposure leads to ER stress-associated cell death in mouse skin

**Fig. 6:**
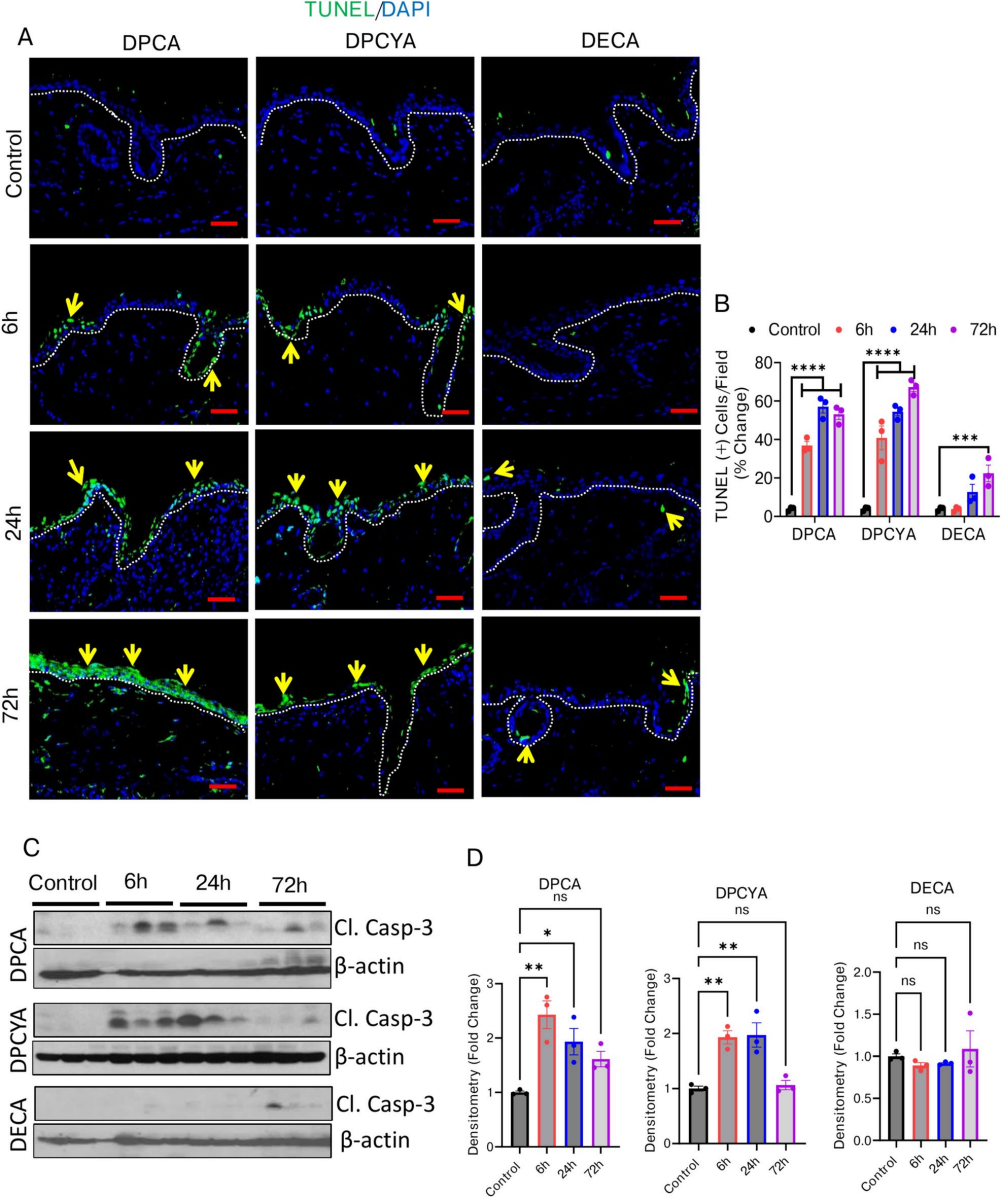
Effects of structurally different arsenicals on apoptotic cell death. (**A**) TUNEL assay of skin sections from vehicle- or treated mice revealed abundant TUNEL-positive (green) cells in DPCA and DPCYA-treated skin, indicating robust cell death (Yellow arrows) at 24 and 72 h. Scale bar-50 µm. (**B**) Histogram showing quantitative analysis of TUNEL-positive green cells. Keyence Microscope inbuilt software (Model BZ-X710, KEYENCE, Osaka, Japan) was used for quantitative analysis of TUNEL-positive cells. Multiple microphotographs captured at 10X magnification was used for quantitative analysis. (**C**) Western blot analysis shows augmented expression of cleaved caspase 3 in arsenical-challenged mice. β-actin was used as an endogenous control. (D) Histogram representing densitometry analysis of western blots band intensity. (Also see supplementary fig. S4, S5 and S6 for full images of immunoblots). *P < 0.05, **P < 0.01,***P < 0.001, ****P < 0.0001 showing significance compared to vehicle treated controls. ns, non-significant. N = 3/group.

So far, our data revealed that exposure to DPCA and DPCYA results in increased expression of the ER stress-regulated transcription factors ATF4 and CHOP. Increased CHOP expression has been linked to cellular apoptosis under elevated stress levels^39^. Therefore, we used the TUNEL assay to determine whether cutaneous exposure to DPCA and DPCYA induces apoptosis in mouse skin. A progressive increase in the number of TUNEL-positive epidermal cells was observed after 6 h, 24 h, and 72 h following exposure to DPCA, DPCYA, and DECA **(**Fig. 6A**).** Quantitative analysis of TUNEL-positive cells revealed that DECA-challenged mice exhibited fewer TUNEL-positive cells compared to those exposed to DPCA and DPCYA **(**Fig. 6B**).** Caspase-3 is a central executive enzyme in orchestrating cell death, and the detection of cleaved caspase-3 is regarded as a reliable marker for apoptosis^51^. Western blot analysis detected cleaved caspase 3 expression in the skin following exposure to DPCA and DPCYA, confirming their role in the induction of apoptotic cell death **(**Fig. 6C, 6D** and Supplementary Fig. S4, S5 and S6).** However, no significant cleaved caspase 3 was detected in the skin of DECA-challenged mice.

## Discussion

Warfare chemicals are highly toxic and therefore their free access is extremely difficult, particularly due to inadequate laboratory conditions for such a work in the university setting. Therefore, it was required to perform this work within the facility which is fully approved by regulating agencies such as Department of Homeland security and Department of Defense besides others in the US. These approved facilities are highly equipped and provide a safe access to these and similar other chemicals by trained manpower to conduct exposures on behalf of the principal investigators and their scientific staff. We performed studies described in this manuscript at MRIGlobal, where following animal exposure and chemical decontamination, rest of the sample analyses were performed at the UAB as also described earlier^20^. This study is a continuation of our investigations related to defining the pathogenesis of lewisite, a powerful arsenical developed during world war I/II for its use as a chemical weapon^19,20,41^. This arsenical is a highly toxic blistering agents which if not decontaminated shortly after its exposure, may lead to lethality^52^. Due to this property, lewisite was given a nick name of “Dew of Death”^53^. We demonstrated that following exposure to murine skin it leads to rapid onset of inflammatory response, which can be observed as erythema and edema development and is followed by tissue disruption leading to necrotic wounds^18^. We also observed a significant increase in tissue chemokine and cytokine levels and skin cell death by apoptosis^41^. Our data identified many commonly upregulated inflammatory genes by these arsenicals such as Csf3, Cxcr2, Ccr1, Tnfsf14, Chi3l3, Cxcl5, Ptgs2 etc*.* Out of these inflammatory genes CSF3, also recognized as granulocyte colony stimulating factor (G-CSF), can potentially contribute to tissue damage by stimulating the bone marrow to produce excessive neutrophils. When these neutrophils are present in higher number, they can release damaging molecules and contribute to tissue destruction^54^. Our data also indicate a significant high infiltration of neutrophils associated with necrosed tissue at 72 h (Fig. 2D and 3B). It has been shown that Cxcl5 activates Cxcr2 in nociceptive sensory neurons to trigger TRPA1 activation driven pain and inflammation^55^. Thus, Cxcl5 and Cxcr2 may underlie the arsenicals-induced pain and nociceptive responses in the skin. Ccr1 and Ptgs2 are identified as key markers that were strongly upregulated after skin injury^56,57^. Similar to these studies, we also found high expression of Ccr1 and Ptgs2 in DPCA and DPCYA exposed mouse skin. The molecular mechanism underlying these changes in the skin involved activation of UPR signaling which is a consequence of protein unfolding and misfolding and leads to protein translational block by the activation of a transcription factor known as ATF4^20,37^. We showed that ATF4 leads to activation of various kinases that in turn phosphorylate eIF2a which underpins protein translational block. We also showed an involvement of ER stress regulated stress granules which by entrapping mRNA block protein translation^19^. The major requirement for these studies was to develop an effective mechanism based MCM. However, it is difficult to develop and stockpile many specific antidotes showing efficacy against only a single arsenical. Therefore, the objective of this study as described in this manuscript was to understand the cutaneous damage caused by these chemicals and define whether similar mechanism underlies the pathogenesis of these chemicals that we observed for lewisite. This provides a likelihood that a common MCM may be effective against a variety of these and other similar additional chemical weapons.

Indeed, we found that most of these chemicals are highly potent skin irritants and cause inflammatory and tissue disrupting response similar to lewisite. We also found an overlap in the alterations of inflammatory genes with the profile of cytokine and chemokines induced by lewisite^19,41^. Interestingly, we observed that majority of these effects are manifested by the augmented oxidative stress caused by the enhanced ROS generation in the skin, which leads to disruption of tight and adherens junctions of the skin epithelial cells in addition to damage basement membrane **(**Fig. 5**)**. This mechanism is involved in the formation of micro-blisters in murine skin. This pattern of gross skin injury and associated molecular disruption is similar to PAO and lewisite^18,41^. Additionally, the molecular mechanism orchestrating these manifestations also seems to be similar to that of lewisite as we found enhanced expression of ATF4 and CHOP which are the major transcription factor of UPR signaling and are consistently induced by all of these arsenicals. In fact, inorganic arsenicals such as ATO also induce these transcription factors^40^. Similar to our earlier studies where we showed that these two transcription factors are involved in inducing apoptosis of the skin keratinocytes and other skin cells, we also found the presence of abundant TUNEL positive epidermal and hypodermal cells. Their numbers increase in a time-dependent manner **(**Fig. 6**)**. This was consistent with the induction of cell death executing cleaved caspase 3. Our data show that the difference between lewisite and these chemicals is the change of severity of skin damage by these chemicals which was in the order: lewisite > DPCA ≥ DPCYA > DECA. This pattern was consistent for both clinical parameters representing injury and mechanistic molecular changes.

The exact cause of variability in the severity of these chemicals is not known. However, there seems to be some structure activity relationship. The organic groups as well as reactive chlorines associated with arsenic in these arsenicals may determine their reactivity, hydrophobicity and thus lipid solubility as well as skin penetration potential. Consistently, while lewisite is diffused rapidly after exposure, these chemicals are relatively slow acting. In addition, changes in their structure also alter their irritation properties^58^. Thus, while lewisite is a major fast acting and highly painful vesicant, the others were developed as sneezing, vomiting and riot-control lacrimating agents^59^. None-the-less they also have potential to cause skin inflammation and blistering.

In summary, we for the first time describe comparative cutaneous pathogenesis of various warfare arsenicals at a molar equivalent dose and discovered a common underlying mechanism by which tissue pathogenesis of these chemicals occur. Based on these data, we believe that the antidote developed for lewisite may also be effective against these chemicals, which needs to be confirmed in further studies. However, these studies are not within the scope of this manuscript.

## Supplementary Information

Supplementary Information.

## Data Availability

All the data supporting the results of this study are available within the paper.
