# Supplementary material for: Common molecular profile of multiple structurally distinct warfare arsenicals in causing cutaneous chemical vesicant injury
Source: Sci Rep. 2025 Feb 22;15:6505. doi: 10.1038/s41598-024-83513-1 (PMC11846883; doi:10.1038/s41598-024-83513-1)
Supplement: Supplementary file 1 — Supplementary Information. [file 41598_2024_83513_MOESM1_ESM.pdf]

MRIGlobal
425 Volker Boulevard
Kansas City, Missouri 64110
Telephone (816) 753-7600
Telefax (816) 753-5519

CERTIFICATE OF ANALYSIS

Project No. 110800.02.002

Diphenylchloroarsine (DA)

Original data is archived under MRIGlobal Project No. 110800.02.002
Serial No. 9194

Compound Identification

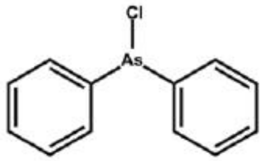

Product:	Diphenylchloroarsine (DA)
IUPAC Chemical Name:	As,As-diphenyl arsinous chloride; Chlorodiphenylarsine
Molecular Formula:	$C_{12}H_{10}AsCl$
Molecular Weight:	264.58
CAS Number:	712-48-1
MRIGlobal Lot No.:	9194-66-07

Quality

Purity:	98.7% by GC-FID
Identity:	Confirmed by NMR and MS analyses
Storage Conditions:	Ambient
Date of Analysis:	February 2, 2016 (GC-FID)
Expiration Date:	No stability testing has been done for this compound

CoA Date

Original Date:	February 19, 2016

Supplementary Figure S1: Certificate of analysis for DPCA purity.

MRIGlobal
425 Volker Boulevard
Kansas City, Missouri 64110
Telephone (816) 753-7600
Telefax (816) 753-5519

CERTIFICATE OF ANALYSIS

Project No. 110800.02.003

Diphenylcyanoarsine (Clark II)

Original data is archived under MRIGlobal Project No. 110800.02.003
Serial No. 9194

Compound Identification

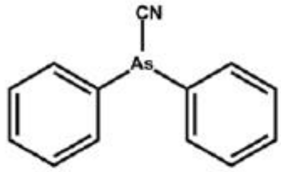

Product:	Diphenylcyanoarsine (Clark II)
IUPAC Chemical Name:	Diphenylarsinecarbonitrile
Molecular Formula:	$C_{13}H_{10}AsN$
Molecular Weight:	255.15
CAS Number:	23525-22-6
MRIGlobal Lot No.:	9194-70-15

Quality

Purity:	99.8% by GC-FID
Identity:	Confirmed by NMR and MS analyses
Storage Conditions:	Ambient
Date of Analysis:	February 5, 2016 (GC-FID)
Expiration Date:	No stability testing has been done for this compound

CoA Date

Original Date:	February 19, 2016

Supplementary Figure S2: Certificate of analysis for DPCYA purity.

MRIGlobal
425 Volker Boulevard
Kansas City, Missouri 64110
Telephone (816) 753-7600
Telefax (816) 753-5519

CERTIFICATE OF ANALYSIS

Project No. 110800.02.004

Diethylchloroarsine

Original data is archived under MRIGlobal Project No. 110800.02.004
Serial No. 9194

Compound Identification

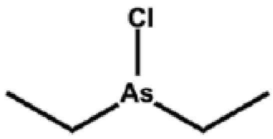

Product:	Diethylchloroarsine
IUPAC Chemical Name:	Diethylarsinous chloride; Chlorodiethylarsine
Molecular Formula:	$C_4H_{10}AsCl$
Molecular Weight:	168.50
CAS Number:	686-61-3
MRIGlobal Lot No.:	9194-67-26

Quality

Purity:	95.6% by GC-FID
Identity:	Confirmed by NMR and MS analyses
Storage Conditions:	Ambient
Date of Analysis:	February 11, 2016 (GC-FID)
Expiration Date:	No stability testing has been done for this compound

CoA Date

Original Date:	February 19, 2016

Supplementary Figure S3: Certificate of analysis for DECA purity.

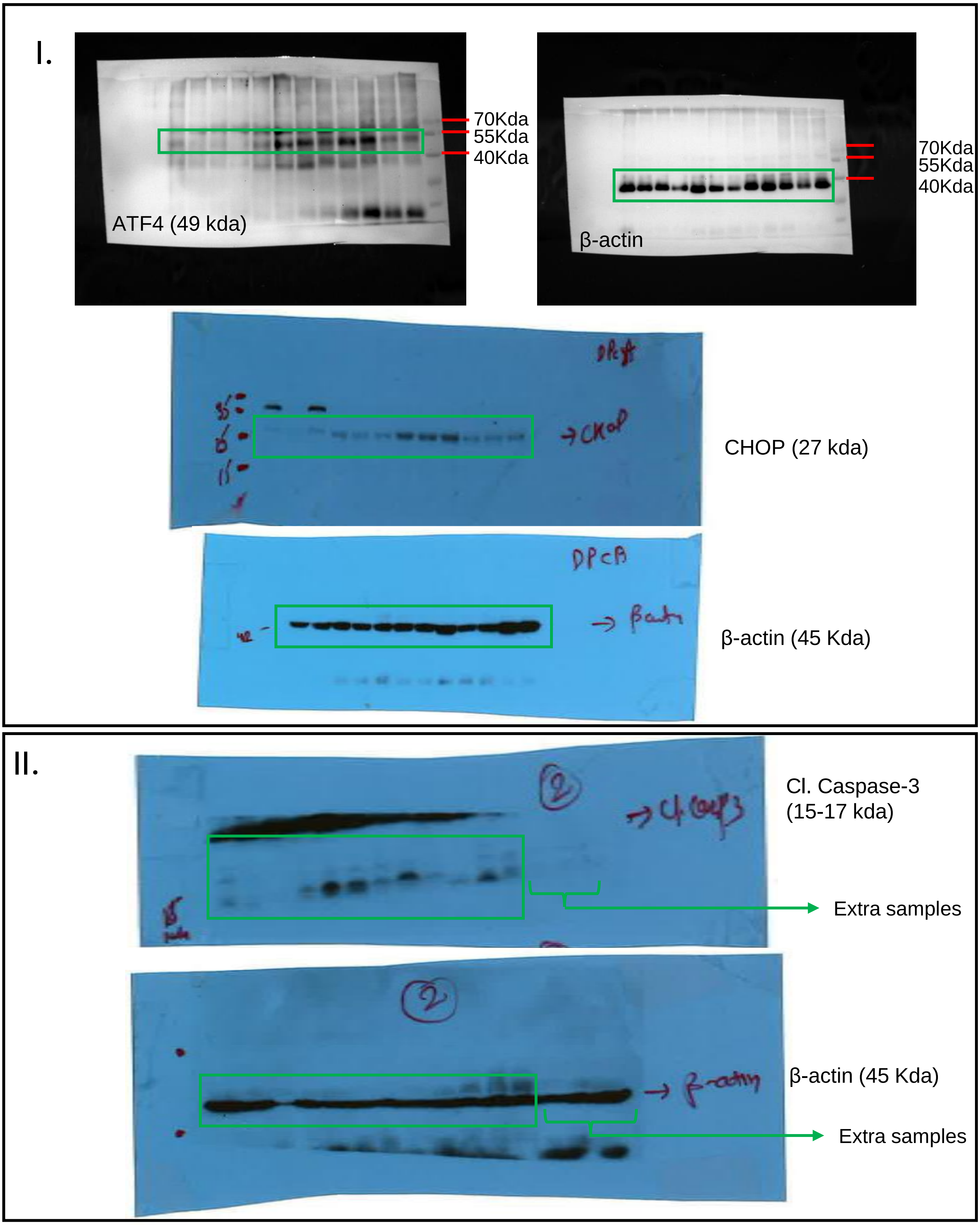

Supplementary Figure S4: Full images of immunoblots related to DPCA chemical presented in Figure 5B (I.) and Figure 6C (II.). Expression of ATF4 protein was detected with an iBright1000 imaging system (Thermo Fisher Scientific, MA, USA), while CHOP and Cl. Caspase-3 expression was detected using Autoradiography film (Densville Scientific Inc.) with Chemiluminescent detection reagent. β-actin was used as endogenous control. Note that the membranes were cut prior to antibodies hybridization.

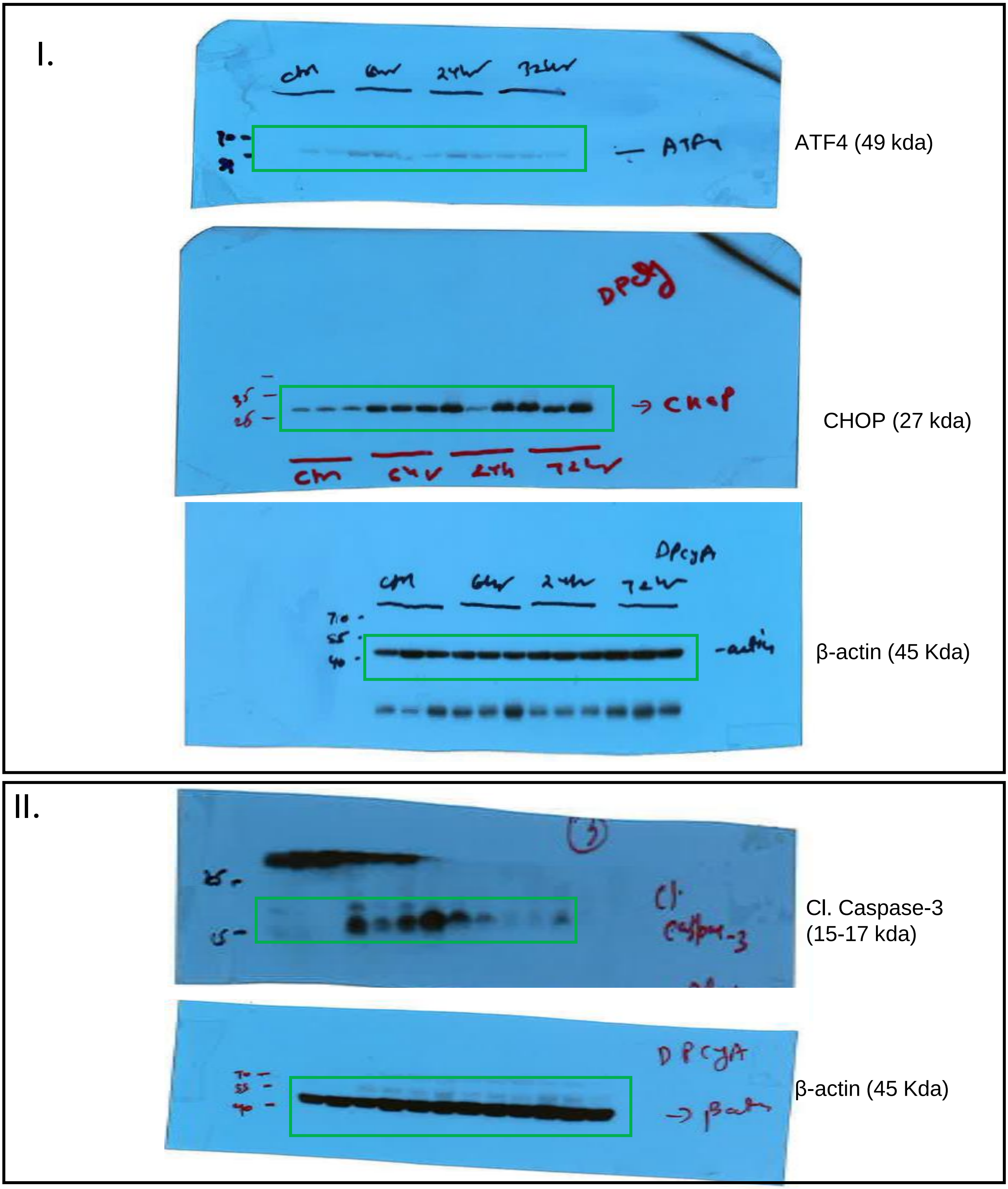

Supplementary Figure S5: Full images of immunoblots related to DPCYA chemical presented in Figure 5B (I.) and Figure 6C (II.). Expression of ATF4, CHOP and Cl. Caspase-3 proteins were detected using Autoradiography film (Densville Scientific Inc.) with Chemiluminescent detection reagent. β-actin was used to detect endogenous control. Note that the membranes were cut prior to antibodies hybridization.

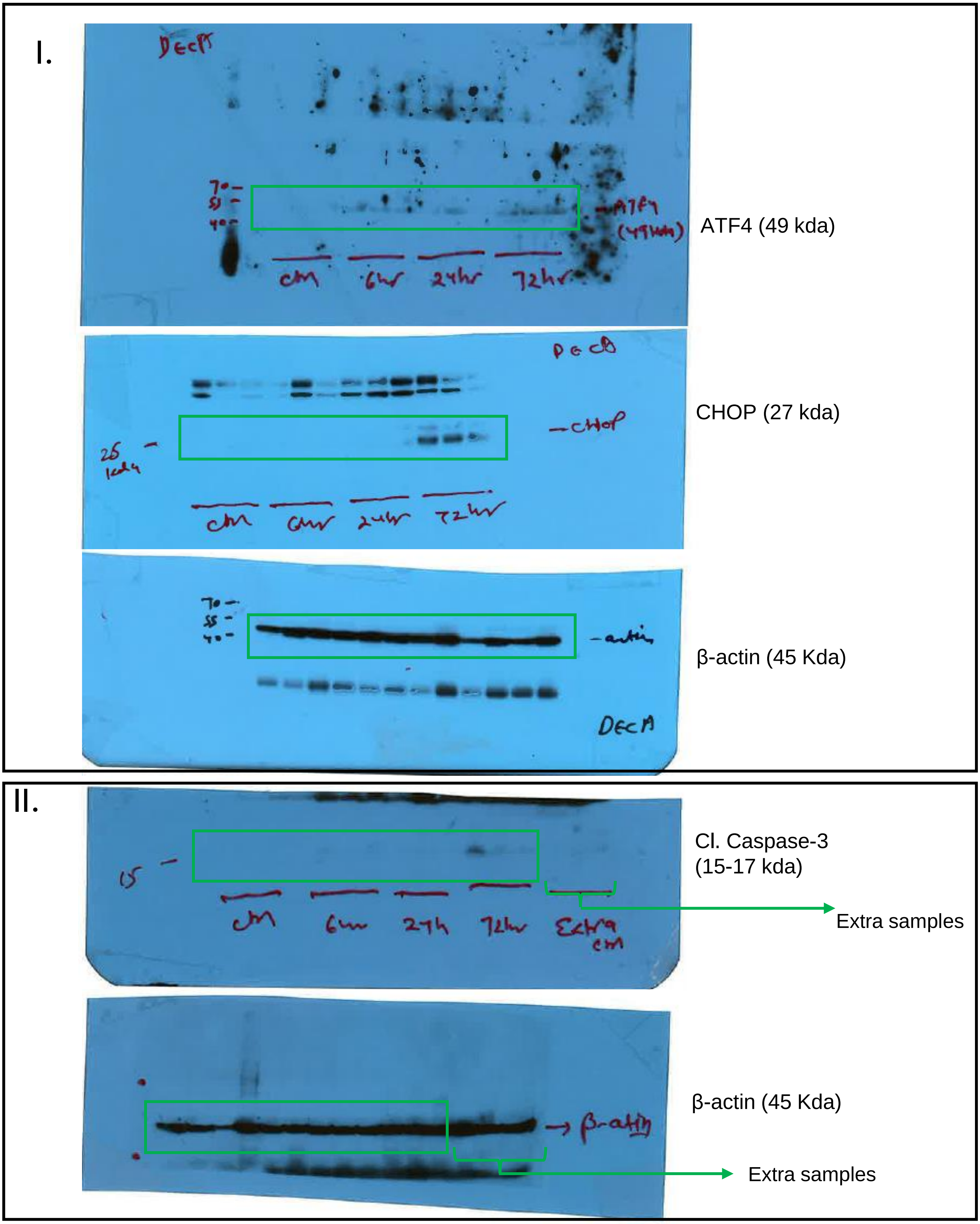

Supplementary Figure S6: Full images of immunoblots related to DECA chemical presented in Figure 5B (I.) and Figure 6C (II.). Expression of ATF4, CHOP and Cl. Caspase-3 proteins were visualized using Autoradiography film (Densville Scientific Inc.) with Chemiluminescent detection reagent. β-actin was used to detect endogenous control. Note that the membranes were cut prior to antibodies hybridization.
